# Comparison of Kinetic and Fluid Simulation Models for RF Capacitively Coupled Plasmas in Semiconductor Processing

**DOI:** 10.3390/ma19091900

**Published:** 2026-05-05

**Authors:** Hwanho Kim, Min Uk Lee, Hae June Lee

**Affiliations:** 1 Department of Electrical Engineering, Pusan National University, Busan 46241, Republic of Korea; hawn93kr@kims.re.kr; 2 Advanced Bio and Healthcare Materials Research Division, Korea Institute of Materials Science, Changwon 51508, Republic of Korea; 3 Division of Semiconductor Engineering, Myongji University, Yongin 17058, Republic of Korea; min.uk.lee@mju.ac.kr

**Keywords:** particle-in-cell simulation, fluid simulation, low-temperature plasma, kinetic effect

## Abstract

As low-temperature plasmas (LTPs) have gained significant attention in materials processing for the microelectronics industry, challenges in spatiotemporal analysis of plasma parameters in a radio frequency capacitively coupled plasma (RF-CCP) system necessitate multidimensional numerical simulations. This study investigated the conditions under which a kinetic simulation or a fluid model is effective for low-pressure CCPs, focusing on the critical role of energy-dependent electron kinetics in LTPs by comparing symmetric and asymmetric electrode structures. We provide a comprehensive investigation of particle energy distributions, elucidating the kinetic effects of non-Maxwellian distributions. The validity of standard fluid approximations, such as the drift–diffusion approximation and isotropic pressure assumptions, is assessed by comparing results from a two-dimensional fluid model with those from a particle-in-cell simulation. The dominance of the ion pressure tensor over isotropic approximations in the sheath has been observed, especially in an asymmetric electrode structure, which is more representative of realistic process chambers.

## 1. Introduction

Low-temperature plasma (LTP) science and engineering fields have witnessed substantial advancements in recent decades [1]. Among the diverse applications of LTPs, materials and chemical processing are the most prominent. Notably, the semiconductor materials processing industry has achieved significant success by harnessing LTPs for critical processes such as etching and deposition. Applications of plasma technology include nanoscale processing, such as atomic layer etching and nanostructure fabrication [2,3,4,5,6,7]. Furthermore, LTP technology has a broad impact on materials processing, including additive manufacturing [8,9,10], soft-material processing, and biomaterial applications [11,12,13,14]. These investigations underscore the multifaceted role of LTP in contemporary science and engineering. While exemplary experimental and theoretical studies have contributed to our understanding of LTPs, parallel numerical modeling research has been pursued to address the challenges of analyzing the spatiotemporal characteristics of plasma systems.

Various simulation schemes have been developed to analyze plasmas, including fluid and particle-in-cell (PIC) simulations, both of which are widely used to analyze LTPs [15,16,17,18,19,20,21,22,23,24]. In a fluid simulation, a set of fluid and Maxwell’s equations is solved [15,16,17,18,19]. The fluid equations are approximately derived from the kinetic equation, as detailed in Section 2. While the kinetic equation is solved for the time evolution of the distribution function in a six-dimensional space of particle position and velocity, the fluid equations are solved for the time evolution of fluid variables in three-dimensional space. Consequently, the fluid simulation requires fewer computational resources compared with the kinetic simulation. Fluid codes are widely used in the study of LTPs due to their relatively straightforward structure and high computational speed, albeit at the cost of accuracy. Another type of plasma simulation is the particle-in-cell (PIC) simulation [20,21,22,23,24]. While a fluid code involves substantial approximations in assuming particle velocity distribution, a PIC code solves the equation of motion for each superparticle trajectory along with Maxwell’s equations. In addition to fully kinetic and fluid approaches, hybrid models have been developed in which the electron component is determined from the Boltzmann kinetic equation in the Lorentz approximation, without additional simplifications to the distribution function [25,26]. Such hybrid models offer intermediate fidelity between fluid and fully kinetic approaches, capturing non-Maxwellian electron kinetics at significantly lower computational cost than PIC simulations.

Collisions in a plasma are demonstrated using Monte Carlo collision (MCC) algorithms. Consequently, the PIC-MCC code captures essential information about particle dynamics, including the minority population of energetic charged particles and their transport, which plays a crucial role in LTPs [27,28,29,30,31]. Despite these advantages, the use of PIC simulations has been limited by the substantial computational cost of tracking a statistically significant number of particles. However, the primary bottleneck of PIC simulations is solving the equation of motion for millions of superparticles, which is inherently suited to massive parallelization as each particle trajectory is computed independently. Modern GPUs exploit this parallelism, with PIC performance scaling nearly proportionally with the number of GPU cores [32], a trend that continues with each hardware generation. As a result, GPU-accelerated PIC simulations have become competitive with multi-CPU fluid simulations [33], substantially lowering the barrier to routine kinetic modeling of LTPs. However, fluid simulations still offer significant benefits in multi-species modeling, as particle-level MCC is still time-consuming. Additionally, not every LTP system requires PIC simulation, depending on the context.

Previous comparisons of PIC and fluid models for CCP discharges have been conducted exclusively in one-dimensional geometries [34,35], establishing that fluid approximations break down at low pressure due to nonlocal electron kinetics and non-Maxwellian distributions. However, these one-dimensional studies cannot address the multidimensional effects arising from geometric asymmetry, such as sidewall-driven ion acceleration and the resulting pressure anisotropy. A systematic two-dimensional comparison of PIC and fluid simulations encompassing both symmetric and asymmetric electrode configurations has not been reported for RF-CCP discharges. Moreover, while previous kinetic studies have focused primarily on electron dynamics [36], the validity of the isotropic pressure closure for ions, which is universally adopted in fluid models, remains unexamined against kinetic simulations in multidimensional geometries.

In this work, we provide guidelines for selecting a suitable simulation model by comparing two-dimensional fluid and PIC simulations of RF-CCPs in symmetric and asymmetric electrode geometries. First, we derive the exact fluid-moment equations from the Boltzmann equation and trace the hierarchy of approximations that lead to the commonly used drift–diffusion and isotropic pressure closures, thereby delineating their formal range of validity. Second, we perform GPU-accelerated electrostatic PIC–MCC simulations and compare them with a fluid code under identical operating conditions to quantify discrepancies arising in low-pressure, nonlocal regimes. Nonlocal electron kinetics refers to the regime in which the electron energy relaxation length $\lambda_{\varepsilon }$ exceeds the characteristic system dimension L. In this regime, electron transport properties are determined by the global potential structure rather than local electric fields, and the electron energy probability function (EEPF) at a given position is influenced by conditions throughout the discharge. Fluid models, which derive transport coefficients from locally defined quantities, inherently cannot capture this behavior. Particular attention is paid to the electron energy probability functions, nonlocal electron temperature profiles, and the full ion and electron pressure tensors in asymmetric reactors, which reveal when kinetic effects render standard fluid approximations inadequate. Finally, based on these results, we provide practical guidance on when fully kinetic or fluid models are required and when advanced fluid models remain sufficient for the modeling of low-temperature plasmas used in materials processing.

In Section 2, accurate fluid equations are derived from the fundamental kinetic equation and reduced to yield the approximated forms used in fluid codes. The approximations used for simplification and their validity are examined in detail. The theoretical analysis [37,38,39] clarifies the explicit limitations of the fluid approach in specific regimes and highlights the conditions under which the PIC method is advantageous. Section 3 presents a comparison of PIC and fluid simulations for capacitively coupled plasmas (CCPs) in symmetric and asymmetric geometries. The simulation results verify the essential features of LTPs that can only be demonstrated by PIC simulations. The kinetic theory elucidates the discrepancies in the fluid simulations. The combined efforts using theory and simulations are summarized in Section 4, where we highlight the importance of collaborative work involving fluid simulations, which remain significant in LTP research.

## 2. Governing Equations and Underlying Theory

Before comparing a PIC simulation with a fluid model, we provide a brief overview of the fluid approximations used in LTPs. We derive and analyze fluid equations from a kinetic perspective, discussing the underlying physical assumptions alongside the exact fluid equations. This analytical derivation provides an explicit evaluation of the validity and applicability of fluid approximations.

### 2.1. Kinetic Analysis

The Boltzmann equation governs the behavior of charged particles in low-temperature plasmas. However, solving the full six-dimensional equation is computationally demanding. To reduce this complexity, fluid models employ a moment expansion of the Boltzmann equation. By taking successive moments of the distribution function, macroscopic transport equations for quantities such as density, momentum, and energy can be derived. In particular, the first-order moment of the Boltzmann equation leads to the momentum conservation equation:

(1) $$mn\left[\frac{\partial u}{\partial t}+(u\cdot \nabla )u\right]=qnE-\nabla \cdot \overset{⃡}{P}+R,$$

where u is the mean velocity, E is the electric field, and R represents the momentum loss due to collisions. Pressure tensor $P_{ij}$ is defined as:

(2) $$P_{ij}=mn{\langle w_{i}w_{j}\rangle }_{v},$$

where $w_{i}=v_{i}-u_{i}$ is the peculiar velocity in the *i*th direction, and ${\left\langle \cdot \right\rangle }_{v}$ denotes the velocity average over the distribution function. In typical fluid models, including this study, the pressure tensor is assumed to be isotropic and thus simplified as a diagonal tensor:

(3) $$\overset{⃡}{P}=\left(\begin{array}{ccc} p & 0 & 0\\ 0 & p & 0\\ 0 & 0 & p \end{array}\right),\;\mathrm{and}\;\mathrm{thus}\;\nabla \cdot \overset{⃡}{P}=\nabla p.$$

The scalar pressure p is then expressed in terms of the particle density and thermal energy.

(4) $$p=\frac{1}{3}mn{\langle {\left|v-u\right|}^{2}\rangle }_{v}$$

To close the system of equations, a thermodynamic equation of state is introduced. Under the assumption of a Maxwellian distribution, an isothermal relation is applied:

(5) $$p=nk_{B}T$$

leading to the following pressure gradient:

(6) $$\nabla p=k_{B}T\nabla n+nk_{B}\nabla T$$

Assuming that the density gradient dominates the temperature gradient, the pressure term is approximated as:

(7) $$\nabla p=k_{B}T\nabla n$$

To derive the drift–diffusion approximation (DDA), we further assume that the inertia term and the convective acceleration term are negligible. Under this condition, the momentum conservation equation [Equation (1)] reduces to a balance between electric force, pressure gradient, and collisional term.

(8) qnE−∇p+R=0

By substituting the collisional term R = *mnν**u*** and assuming a stationary background gas and a constant collision frequency, we derive the final form of the DDA.

(9) Γ=μnE−D∇n

where μ is the mobility, and D is the diffusion coefficient of the species.

### 2.2. Fluid Simulation

For the fluid simulation, we employed the Hybrid Plasma Equipment Model (HPEM) [40]. HPEM offers several options for evaluating electron-impact reaction rates: the electron Monte Carlo Simulation (eMCS), a kinetic approach in which electron trajectories are tracked with Monte Carlo collisions within the fluid framework, and fluid-based approaches such as the local field approximation (LFA) [41]. In this work, we chose to evaluate reaction rates based on $T_{e}$ rather than eMCS to maintain consistency with widely used fluid modeling practice and to isolate the effects of fluid approximations in the comparison with PIC. The time-dependent electron energy equation is obtained by:

(10) $$\frac{\partial }{\partial t}\left(\frac{3}{2}n_{e}k_{B}T_{e}\right)=\nabla \cdot \left(\kappa \nabla T_{e}\right)+\nabla \cdot \left(\Gamma_{e}T_{e}\right)=P$$

where κ is the thermal conductivity, $\Gamma_{e}$ is the electron flux, and

(11) $$P=J\cdot E=q_{e}\Gamma_{e}\cdot E$$

represents the power deposition from the electric field. The electron temperature, defined as two-thirds of the mean electron energy derived from the EEPF, serves as a key parameter for calculating rate constants. The electron flux is assumed to follow the DDA.

This approximation is widely employed in low-temperature plasma modeling [42,43,44,45], where it provides a reasonable balance between physical fidelity and computational efficiency. Although more complete models (e.g., solving the full momentum equation) may offer improved accuracy in capturing transient behaviors, the DDA remains a practical and validated choice for simulating the spatial evolution of electron density and energy in many LTP applications. While DDA is applied to electrons for computational efficiency, the momentum conservation equation is used for ions to capture their transport dynamics.

The distinction between electrons and ions arises from their fundamentally different mass and momentum relaxation times. Due to their small mass and frequent collisions, electrons rapidly reach a quasi-steady-state drift velocity, which justifies the use of the drift–diffusion approximation. In contrast, ions, owing to their significantly larger mass, exhibit finite acceleration and non-negligible inertia, particularly in regions of strong electric fields such as plasma sheaths. Therefore, the full momentum conservation equation is retained for ions to accurately capture their transport dynamics. This modeling strategy is consistent with widely used fluid descriptions of low-temperature plasmas, in which electron transport is often reduced to a drift–diffusion form, whereas ion transport retains finite inertia via the momentum conservation equation.

### 2.3. PIC Simulation

A GPU-parallelized electrostatic PIC simulation is employed in this study, using an in-house code written in C and CUDA [32]. The overall PIC algorithm proceeds in a loop comprising particle mover, MCC, and charge deposition (weighting), followed by solving the Poisson equation to obtain the updated electric field. The motion of charged particles is computed by solving Newton’s equation of motion:

(12) $$m\frac{dv}{dt}=qE$$

where the magnetic field ***B*** is neglected (*B* = 0). Particle collisions with background neutrals are modeled using an MCC algorithm. For each time step, the collision probability $P_{i,k}$ is calculated for a particle with velocity $v_{i}$ and energy $\varepsilon_{i}$ using

(13) $$P_{i,k}=1-exp\left[-n_{g}\sigma_{k}(\varepsilon_{i})v_{i}∆t\right]$$

where $n_{g}$ is the neutral gas density, and $\sigma_{k}$ is the energy-dependent cross-section for the *k*-th collision. While both fluid and PIC methods solve the same electrostatic equations, the PIC method is distinguished by its direct simulation of particle motion.

## 3. Comparison of PIC and Fluid Simulations

**Figure 1:**
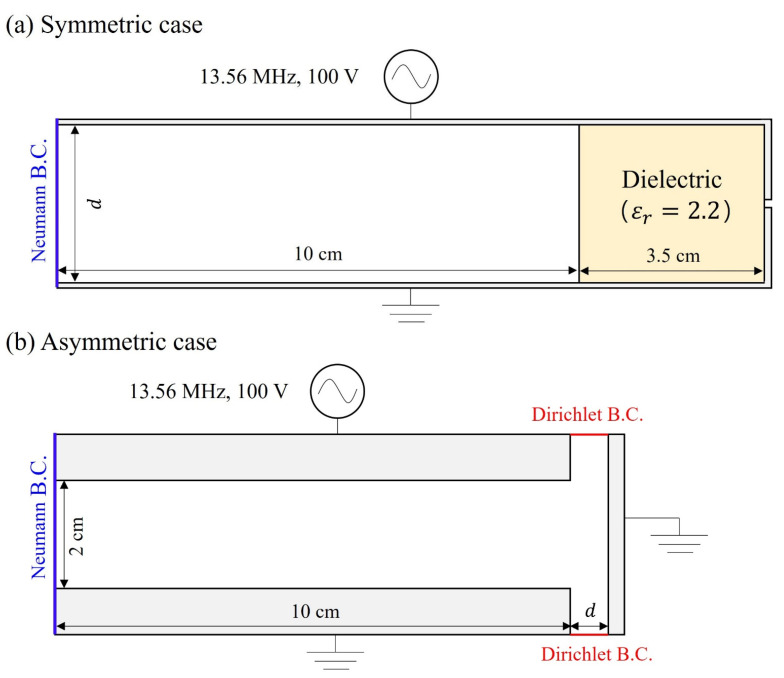
Schematic diagrams of the simulation domain: (**a**) a symmetric geometry and (**b**) an asymmetric geometry.

Figure 1 shows the symmetric and asymmetric structures used for both PIC and fluid simulations. In the symmetric configuration (Figure 1a), the powered top electrode and grounded bottom electrode have equal surface areas. To suppress sidewall effects, a dielectric material with a thickness of 3.5 cm and a relative permittivity ($\varepsilon_{r}$) of 2.2 is inserted. This configuration is specifically designed to mimic a one-dimensional (1D) geometry closely. In contrast, the asymmetric case (Figure 1b) includes a sidewall, introducing a strong electric field due to the difference in electrode area.

The numerical conditions are summarized in Table 1. The gap distance (*d* in Figure 1) refers to the electrode spacing in the symmetric case and the sidewall spacing in the asymmetric case. For argon, electron collisions include elastic scattering, excitation (treated as a single effective cross-section), and ionization, using the Phelps database [46]. Ion–neutral collisions include momentum transfer and charge exchange. Identical collision cross-section data are used in both models.

Using a GPU-accelerated 2D PIC code, we capture the pressure tensor effect and associated forces that are not fully resolved by fluid simulations. To compare results, we analyze spatial profiles of steady-state CCPs averaged over 10 RF cycles. The spatial and temporal resolutions satisfy the CFL condition, and the number of superparticles ensures convergence. Despite the smaller time step and the finer grid resolutions in the PIC simulation, the total computation time (~12 h) was comparable between the GPU-PIC (RTX 3090) and the fluid simulation (Xeon E5-2697 v2, 12 cores).

We consider the steady state to be reached when key plasma parameters, such as the total particle numbers and the density profiles, exhibit periodic convergence over successive RF cycles within a specified tolerance. Although a direct hardware comparison is not straightforward given the different platforms, this result illustrates that GPU-accelerated PIC has reached a level of practical feasibility for routine 2D LTP analysis, with further improvements expected as GPU architectures continue to advance.

### 3.1. Symmetric Structure

**Figure 2:**
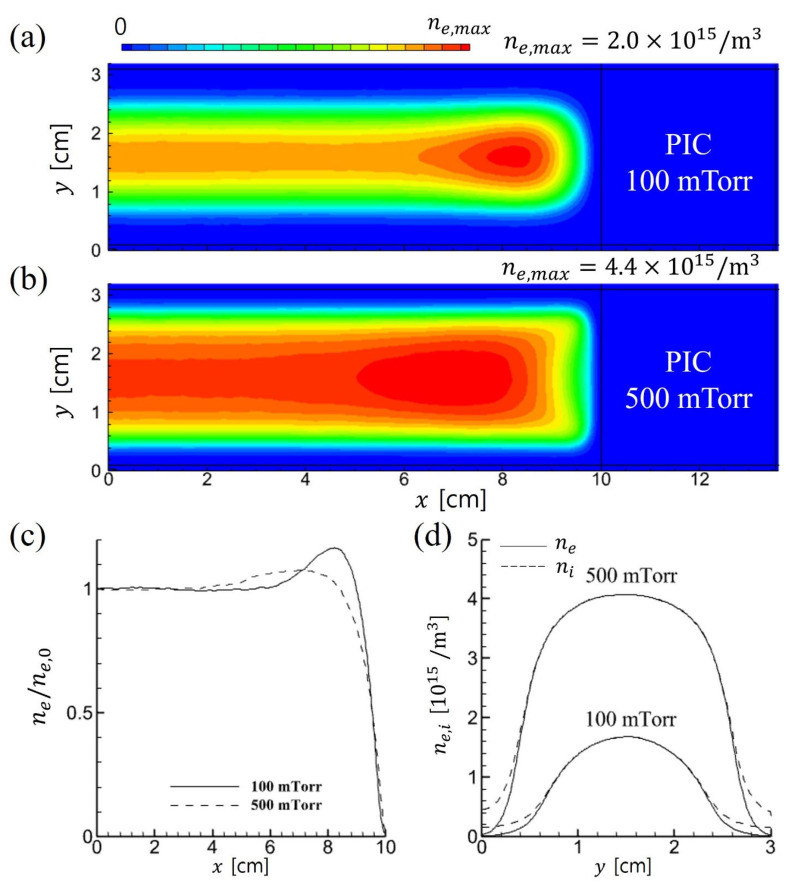
Time-averaged 2D profiles of electron density in PIC simulations for (**a**) 100 mTorr and (**b**) 500 mTorr. 1D profiles are extracted at (**c**) *y* = 1.5 cm in the horizontal direction, and at (**d**) *x* = 0.5 cm in the vertical direction.

**Figure 3:**
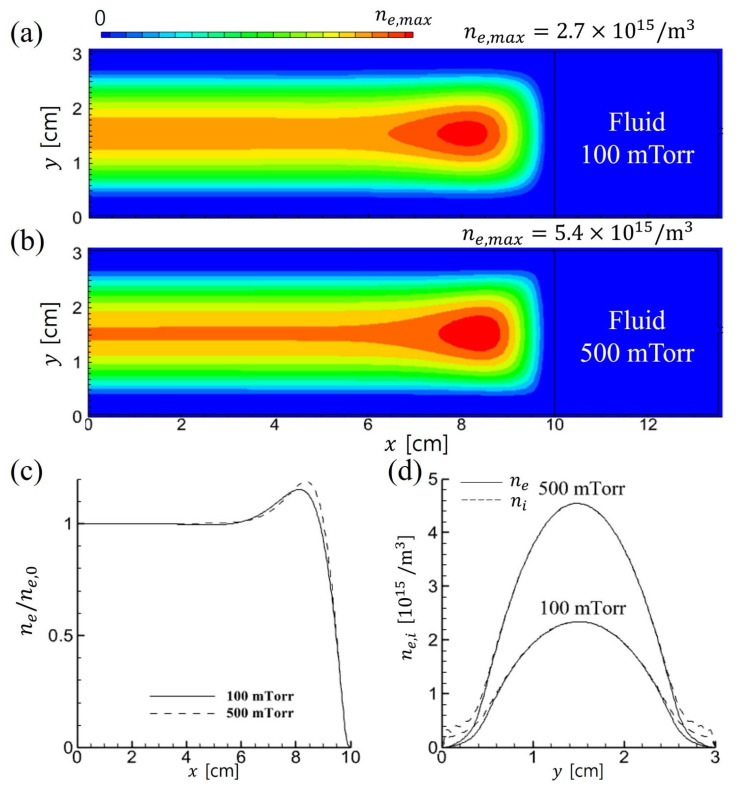
Time-averaged 2D profiles of electron density in fluid simulations for (**a**) 100 mTorr and (**b**) 500 mTorr. 1D profiles are extracted at (**c**) *y* = 1.5 cm in the horizontal direction, and at (**d**) *x* = 0.5 cm in the vertical direction.

A comparison of the 2D electron density distributions is shown in Figure 2 (PIC) and Figure 3 (fluid). In both simulations, the electron density increases with gas pressure because the higher neutral density enhances the ionization rate. However, only the PIC simulation exhibits an enhancement in the uniformity of the density profile and an expansion of the bulk plasma region as pressure increases.

**Figure 4:**
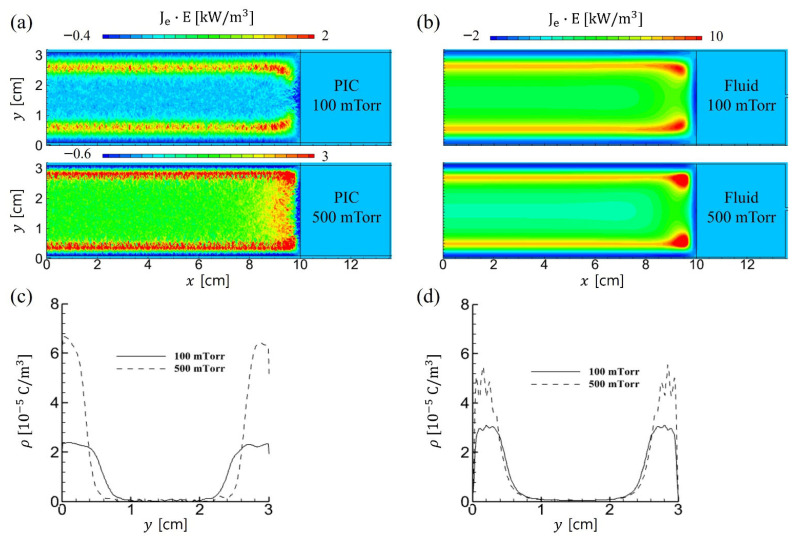
Time-averaged 2D profiles of electron power absorptions in (**a**) PIC and (**b**) fluid simulations for 100 mTorr and 500 mTorr. 1D profiles of the time-averaged space charge density are measured at *x* = 0.5 cm in the vertical direction for (**c**) PIC and (**d**) fluid simulations.

The electron power absorption profiles for both simulation methods are nearly identical, as shown in Figure 4a,b, which depict broadened bulk plasma in the y-direction with increasing gas pressure. Figure 4c,d show the space charge density for both simulations. At 500 mTorr, the PIC results show a more pronounced increase in space charge density and a greater reduction in the sheath width compared with the fluid simulation. This stronger electric field accelerates sheath electrons toward the plasma bulk during the sheath expansion phase, driven by the oscillating external voltage source. The intensified electric field also promotes stronger stochastic heating near the bulk-sheath boundary, generating energetic electrons that enhance ionization there and help sustain the broader bulk plasma.

**Figure 5:**
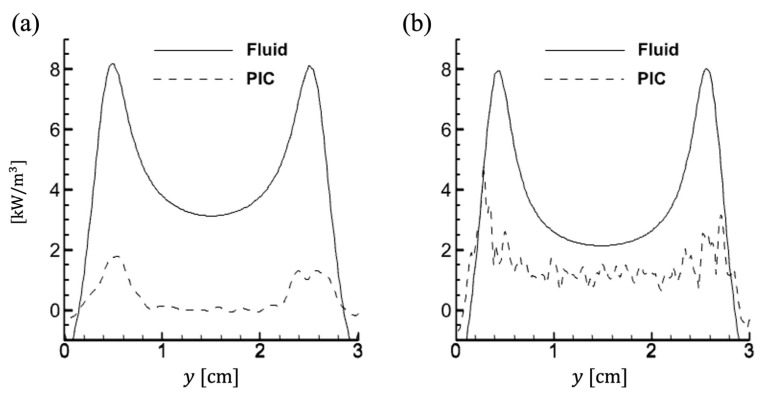
Time-averaged 1D profiles of electron power absorption are extracted at *x* = 0.5 cm in the vertical direction for (**a**) 100 mTorr and (**b**) 500 mTorr.

In contrast, notable differences appear in the bulk region. Figure 5 presents the electron power absorption profiles along the y-direction near the center. Overall power absorption is higher in the fluid simulation than in the PIC simulation. Moreover, Ohmic heating in the bulk plasma for the PIC simulation (dashed lines) is negligible at 100 mTorr but becomes significant at 500 mTorr. On the other hand, the fluid results (solid lines) show noticeable ohmic heating at both pressures. Similar trends have been reported in previous 1D simulation studies [47]. This discrepancy arises from the use of the DDA to solve the electron energy equation (Equation (10)), in which transport properties depend only on the electric field and diffusion. Since this assumption holds better at higher pressures, the electron flux in low-pressure regimes is overestimated, leading to electron power absorption ($J_{e}\cdot E$) in the bulk that resembles high-pressure behavior. It proves that the DDA is not appropriate for low-pressure RF discharges.

**Figure 6:**
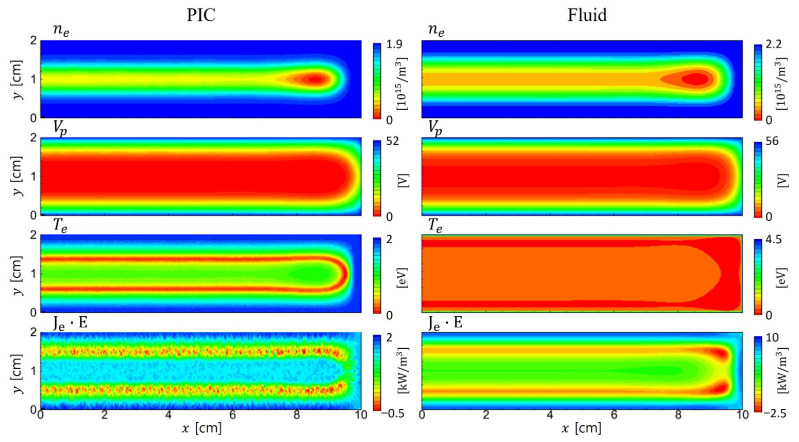
Time-averaged 2D profiles of electron density, potential, electron temperature, and electron power absorptions in PIC (**left**) and fluid (**right**) simulations for the symmetric electrodes.

**Figure 7:**
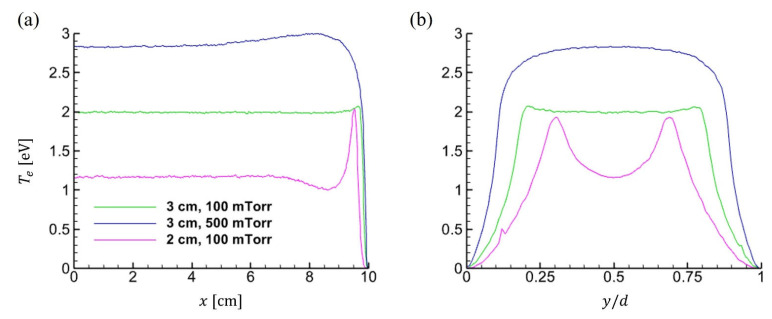
Electron temperature profiles with changing *pd* values. Time-averaged 1D profiles are extracted at (**a**) *y* = 1.5 cm in the horizontal direction, and at (**b**) *x* = 0.5 cm in the vertical direction. In Figure (**b**), *y/d* represents the *y*-axis normalized to the gap distance.

Figure 6 compares the time-averaged electron density, plasma potential, electron temperature, and power absorption profiles for the PIC simulation (left column) and the fluid model (right column). While electron density, potential, and electron power absorption quantities exhibit similar trends, a significant discrepancy is observed in the electron temperature profiles. In the PIC results, a local minimum in the electron temperature is observed within the bulk region, as shown in Figure 7.

This local drop in temperature appears near *x* = 8.5 cm in Figure 7a and at the gap center (*y/d* = 0.5) in Figure 7b, particularly for the 2 cm gap. This temperature profile cannot be obtained from the fluid simulation. Finally, it was shown that the fluid model introduces errors in nonlocal electron kinetics. Similar phenomena have been reported in previous studies [47,48] and are attributed to nonlocal electron kinetics in low-pressure, collisionless regimes. In such regimes, electron transport is governed by individual kinetic energy and the local electric potential rather than by electron-neutral collisions. As a result, electrons conserve their total energy as they oscillate within the ambipolar potential formed by the presheath. Low-energy electrons, whose kinetic energy is insufficient to overcome the sheath potential, remain confined within the bulk plasma.

In contrast, high-energy electrons can reach the sheath edge and contribute to stochastic heating. This results in the local extrema in the electron temperature profile near the sheath boundary. Fluid models, which assume quasi-Maxwellian distributions sustained by frequent collisions, inherently fail to capture these kinetic effects, yielding smoother but less accurate temperature profiles under such conditions. Despite the discrepancy in electron temperature, all other profiles in Figure 6 are in good agreement between the two simulations. It is also due to electron nonlocal kinetics, which extends the energy relaxation length and thus primarily affects the plasma density profiles via diffusion.

**Figure 8:**
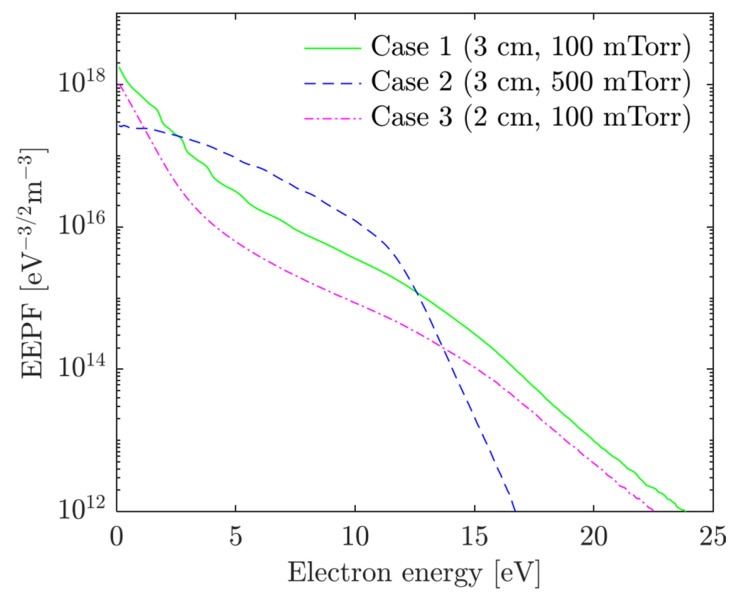
EEPF at the center of the gap for the cases 1–3.

Figure 8 presents the EEPF for cases 1–3, corresponding to increasing *pd* values in the order: case 3 < case 1 < case 2. As *pd* increases, the EEPF evolves from a bi-Maxwellian shape (case 3) to a quasi-Maxwellian (case 1), and finally to a Druyvesteyn distribution (case 2). The EEPF in case 3 shows a clear separation: low-energy electrons ($\varepsilon_{e}$ < 5 eV) are confined by the ambipolar potential, while higher-energy electrons ($\varepsilon_{e}$ > 5 eV) reach the sheath edge and undergo stochastic heating. In case 1, this separation becomes less distinct, indicating a partial transition toward collisional behavior. Case 2 exhibits a qualitatively different profile. The elevated momentum transfer collision frequency leads to significant Ohmic heating in the bulk plasma, as evidenced by Figure 4 and Figure 5. This mechanism increases the energy of low-energy electrons (e.g., *ε* < 11 eV), broadening the distribution. However, above the inelastic threshold energy (e.g., 11.55 eV for excitation), inelastic collisions dominate, causing a sharp drop in the EEPF beyond 11 eV.

**Figure 9:**
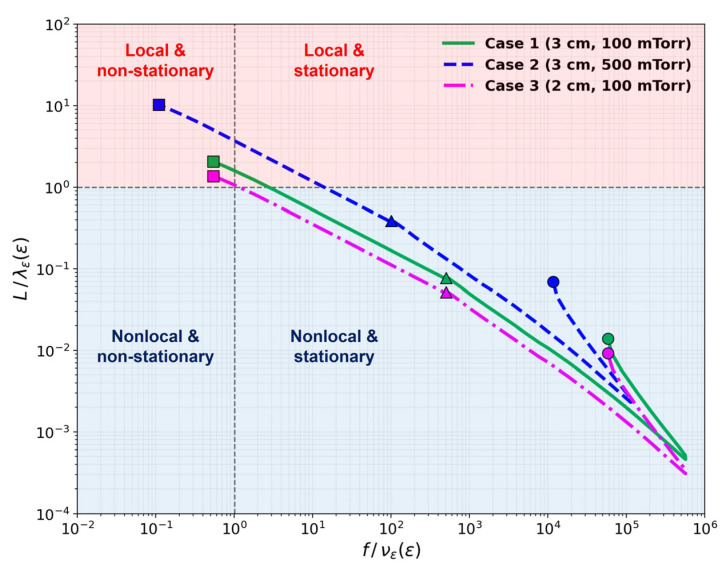
Electron energy relaxation regime map for ($f/\nu_{\varepsilon }$, $L/\lambda_{\varepsilon }$) parameter space for cases 1–3. Each curve traces the energy dependence from thermal energies (circles) to inelastic threshold energies (squares for ionization and triangles for excitation).

To provide a quantitative criterion for the validity of fluid approximations, Figure 9 maps each simulation case onto ($f/\nu_{\varepsilon }$, $L/\lambda_{\varepsilon }$) parameter space, where f is the RF frequency, $\nu_{\varepsilon }$ is the energy relaxation frequency, L is the electrode gap, and $\lambda_{\varepsilon }$ is the electron energy relaxation length [49]. Each curve traces the locus of a single discharge condition as the electron kinetic energy is swept from 0.026 eV to 15.8 eV (ionization threshold), with circle (○), triangle (△), and square (□) markers indicating ε= 0.026 eV, 11.5 eV (excitation threshold), and 15.8 eV (ionization threshold), respectively. While low-energy bulk electrons in all three cases reside in the nonlocal regime ($L/\lambda_{\varepsilon }<1$), electrons above the ionization threshold (~15.8 eV) transition into the local regime ($L/\lambda_{\varepsilon }>1$).

The change in the EEPF by the variation in the product of gas pressure and gap distance, *pd*, is related not only to the ratio of the gap distance to the energy relaxation length, $L/\lambda_{\epsilon }$, but also to the ratio of the driving frequency to the energy relaxation frequency, $f/\nu_{\epsilon }$ [49]. If $L/\lambda_{\epsilon }$ < 1, nonlocal kinetics is dominant and the bi-Maxwellian EEPF is obtained. This energy-dependent locality is central to the discrepancies observed in fluid models: the ionization and excitation rate constants are determined predominantly by the high-energy tail of the EEPF, which is governed by nonlocal transport regardless of the bulk collisionality. When $f/\nu_{\epsilon }$ < 1, the electron energy relaxation is very fast, and the isotropic term of the EEPF is not stationary. Thus, the time-derivative term is dominant in this case, yielding the bi-Maxwellian EEPF [49].

### 3.2. Asymmetric Structure

**Figure 10:**
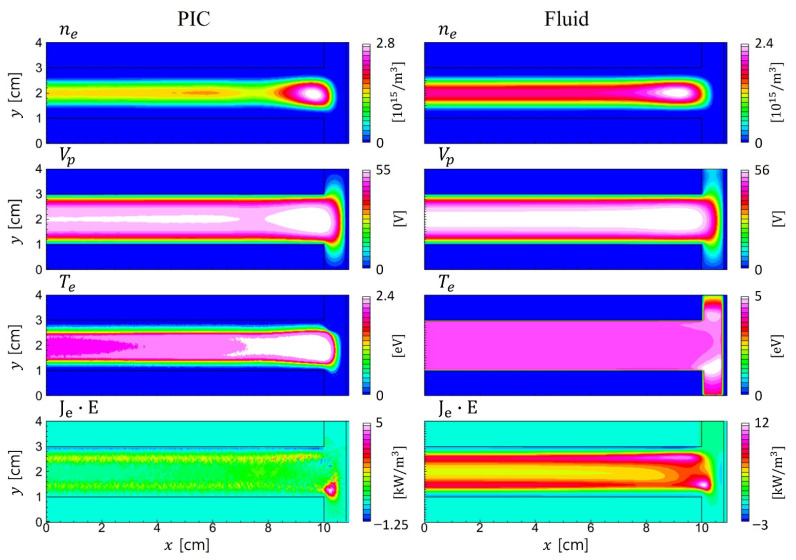
Time-averaged 2D profiles of electron density, potential, electron temperature, and electron power absorptions in PIC (**left**) and fluid (**right**) simulations for the asymmetric electrodes.

**Figure 11:**
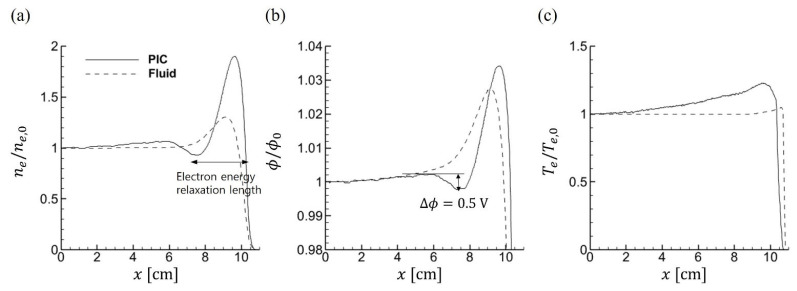
Normalized (**a**) electron density, (**b**) plasma potential, (**c**) electron temperature profiles in the asymmetric domain, measured at *y* = 2 cm in the horizontal direction. All values are normalized with respect to the center value (*x* = 0).

The asymmetric structure introduces kinetic behaviors that differ fundamentally from those in symmetric configurations. Figure 10 shows that, in addition to discrepancies in electron temperature, the asymmetric case exhibits marked spatial nonuniformity between the PIC and fluid simulations. As shown in Figure 11, a local minimum in electron density forms along with a corresponding potential well. These extrema appear only in the PIC simulation, while the fluid model yields a broader, smoother profile without localized features. This behavior arises because the electron energy relaxation length depends on kinetic energy [48,50]. In the PIC model, energetic electrons with energies exceeding the ionization threshold energy (15.76 eV) are accelerated near the sidewall, where they induce ionization locally and form a peak in electron density.

**Figure 12:**
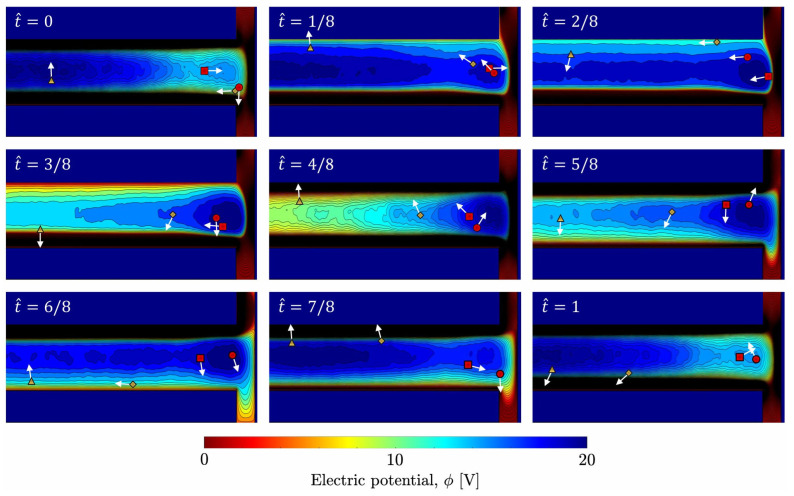
Trajectories of sampled electron superparticles (○, □: trapped electrons; △, ◇: untrapped electrons) at different RF phases ($\hat{t}=tf_{RF}$) for *d* = 0.8 cm at 100 mTorr. The background color map represents the time-varying electric potential. Arrows indicate the velocity direction of each particle.

In contrast, low-energy electrons with longer relaxation lengths traverse a wider region without contributing to ionization. This results in a nonuniformity in electron density between the wall and the central plasma region. The time-averaged electron temperature increases from the center toward the edge, indicating enhanced electron heating near the sidewall. In addition, the PIC results reveal localized trapping of low-energy electrons in regions where the potential exhibits a local maximum [51,52]. This energy-dependent trapping, characteristic of kinetic transport, cannot be captured by fluid models and contributes to the formation of spatial nonuniformities in the electron distribution. Figure 12 illustrates the trajectories of sampled electron superparticles over one RF cycle in the asymmetric geometry with *d* = 0.8 cm at 100 mTorr. Trapped electrons (○, □) oscillate within the potential well, whereas untrapped electrons (△, ◇) traverse the domain with sufficient kinetic energy. This visualization, accessible only through PIC simulations, directly demonstrates the energy-dependent trapping mechanism described above.

Beyond the nonlocal and nonlinear behaviors discussed earlier, our results reveal that the plasma also exhibits pressure anisotropy due to non-negligible ion viscosity. Although the ion momentum equation is solved in fluid models, these models typically assume isotropic pressure and neglect shear-stress effects. In contrast, the PIC results presented here reveal that off-diagonal components such as $P_{xy}$ can reach up to 10% of the diagonal terms. It indicates the importance of off-diagonal components of the pressure tensor in asymmetric plasma conditions. This observation corresponds to a case with a 3 cm sidewall gap, as discussed below.

As to the comparison of EEPFs, Figure 8 of Reference [50] shows the changes in the low- and high-energy regimes of the EEPFs at different positions: the center, the density minimum, and the density peak for the nonlocal electron kinetics at 100 mTorr. The long electron energy relaxation length allows electrons to travel without energy loss, producing a bi-Maxwellian EEPF. However, strong electron heating and increased low-energy temperature are observed at the density minimum. It means that electron transport has been enhanced, but ionization remains low. On the contrary, the sidewall heating is strong at the density-peak position, where the high-energy tail of the EEPF is also strong.

**Figure 13:**
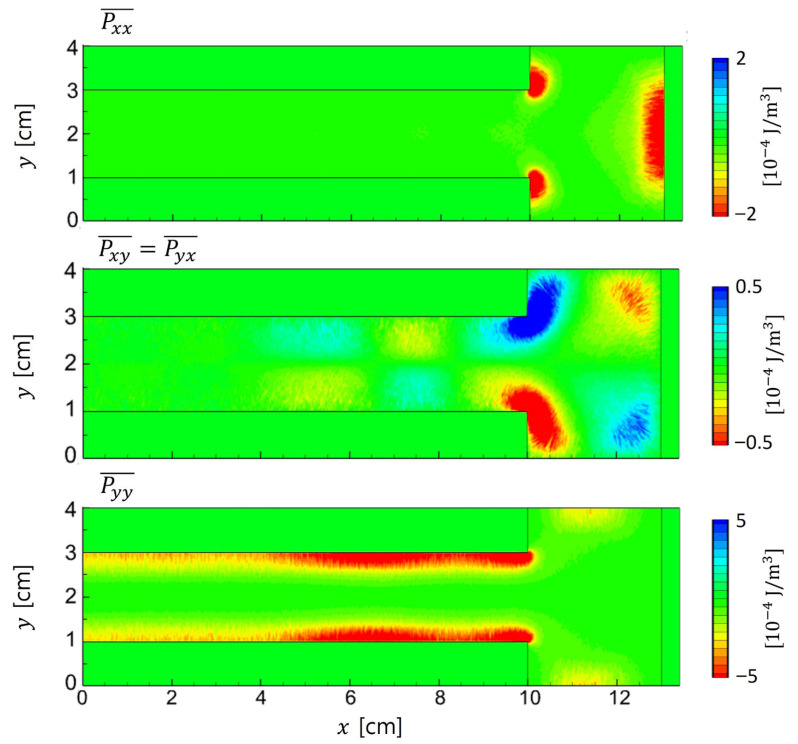
Spatial components of the ion pressure tensor $\overset{⃡}{P_{i}}$ obtained from PIC simulation with asymmetric electrodes. Note that by definition $\overset{⃡}{P_{i}}=m_{i}\int w_{i}w_{i}f_{i}dv$, the pressure tensor is symmetric ($P_{ij}=P_{ji}$).

**Figure 14:**
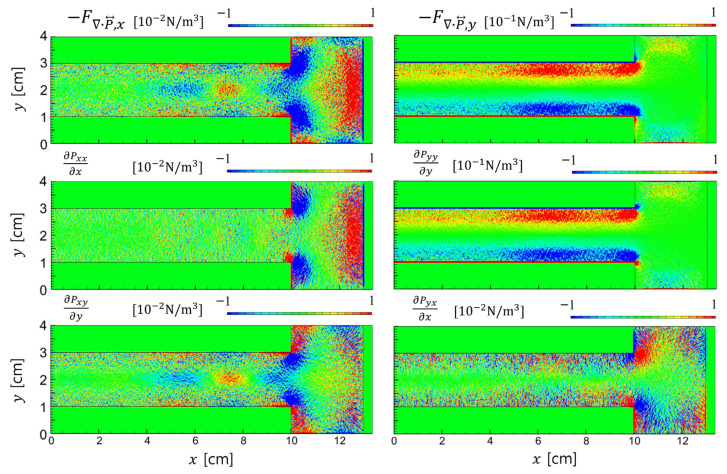
Spatial components of $\nabla \cdot \overset{⃡}{P_{i}}$, the divergence of the ion pressure tensor associated with the diffusion force, obtained from PIC simulation with asymmetric electrodes.

As shown in Figure 13, the ion pressure tensor exhibits distinct spatial variation across regions. Within the sheath, where the electric field primarily drives ion motion, the diagonal components are dominant. In this regime, the influence of off-diagonal terms remains negligible, supporting the use of isotropic approximations. However, in the bulk plasma region for 4 < *x* < 10 cm, the off-diagonal components become comparable in magnitude to the diagonal terms. These off-diagonal components alternate in sign across space and significantly influence the divergence term $\nabla \cdot {\overset{⃡}{P}}_{i}$ as detailed in Figure 14.

The relative discrepancy between the realistic forces (top row) and the approximated forces with the isotropic pressure (second row) is more severe in the *x*-direction than in the *y*-direction. It means that the 1D PIC simulation cannot account for the effects of the anisotropic pressure tensor. The anisotropic pressure contributions (third row) primarily arise in the sheath regime, including the plasma-material interface. However, the *x*-directional force is very strong in the bulk plasma for 4 < *x* < 10 cm, which holds significant importance in LTPs. It is worth noting that the asymmetric geometry discussed in this section is a simplified example of a process equipment configuration. In realistic plasma equipment with complex geometries and various dielectric materials, PIC simulations remain essential for the precise analysis of LTPs.

**Figure 15:**
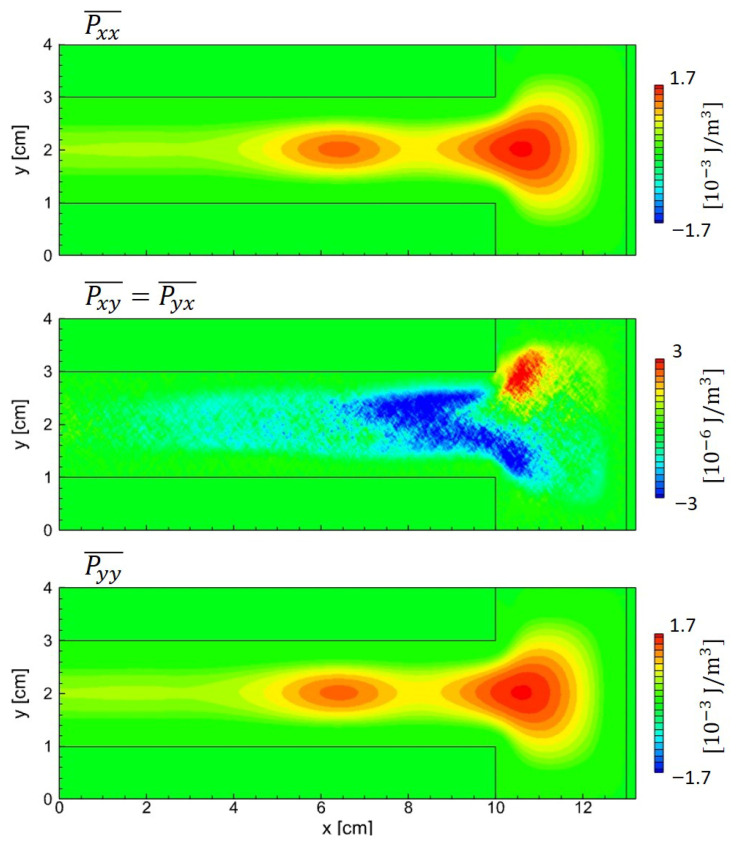
Spatial components of the electron pressure tensor $\overset{⃡}{P_{e}}$ obtained from PIC simulation with asymmetric electrodes.

**Figure 16:**
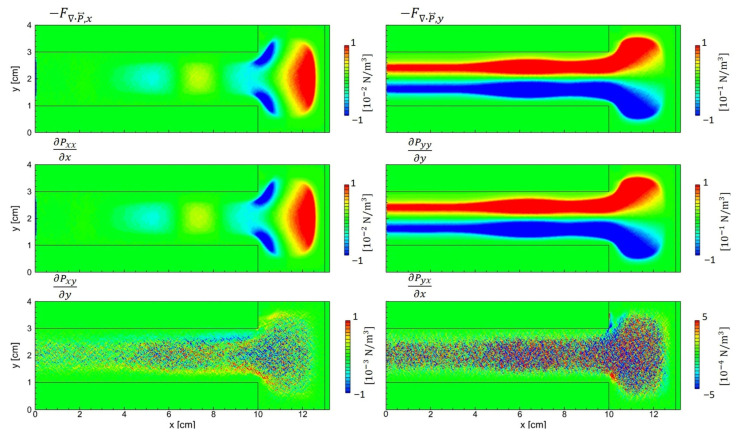
Spatial components of $\nabla \cdot \overset{⃡}{P_{e}}$, the divergence of the electron pressure tensor associated with the diffusion force, obtained from PIC simulation with asymmetric electrodes.

The electron pressure tensor exhibits distinct behavior, as shown in Figure 15. $P_{xx}$ and $P_{yy}$ are nearly identical in magnitude and spatial dependence, whereas $P_{xy}$ exhibits a different spatial distribution and is smaller than the diagonal components by about two to three orders of magnitude. This contrast is consistent with the electron heating sequence [36]. During the expansion and collapse of the sidewall sheath, the electron velocity distribution becomes anisotropic, whereas momentum transfer collisions in the bulk isotropize the electron distribution. This regime holds when the energy relaxation frequency ($\nu_{\varepsilon }$) greatly exceeds the RF field frequency (ω), $\nu_{\varepsilon }\gg \omega$, meaning electron energy relaxation is faster than the field oscillation. The divergence term associated with the electron pressure tensor $\nabla \cdot {\overset{⃡}{P}}_{e}$ is shown in Figure 16. The realistic *x*- and *y*-directional forces (top row) are dominated by the gradients of the isotropic pressure (second row). In contrast, the anisotropic pressure contributions (third row) are smaller by about two to three orders of magnitude and therefore negligible for electrons. This behavior contrasts with that in the ion case in asymmetric geometries, where significant shear stresses induce off-diagonal components.

**Figure 17:**
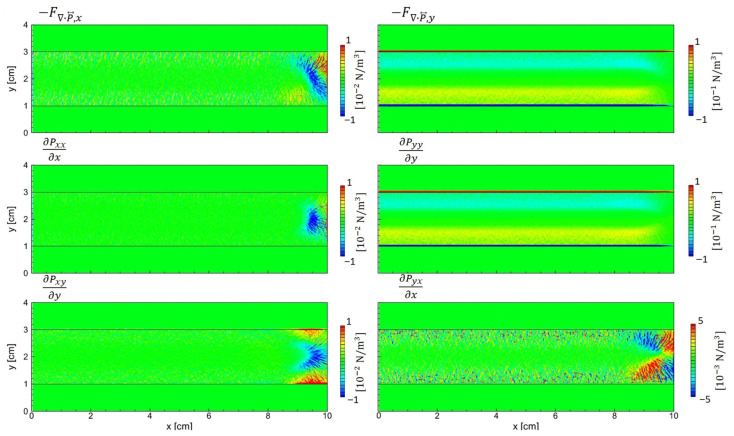
Spatial components of $\nabla \cdot \overset{⃡}{P_{i}}$, the divergence of the ion pressure tensor associated with the diffusion force, obtained from a PIC simulation with symmetric electrodes.

Figure 17 presents the ion pressure-gradient force $\nabla \cdot {\overset{⃡}{P}}_{i}$ in a symmetric geometry. Unlike the asymmetric case, the *x*- and *y*-directional forces are negligible across the bulk region and become appreciable only within the sheaths. The anisotropic pressure tensor terms (third row) in the bulk are essentially absent, since their enhancement relies on an anisotropic velocity distribution driven by the sidewall sheath, which is not present in the symmetric configuration. A previous study [53] showed that the treatment of ion transport, whether modeled using a DDA or the full ion momentum equation, can markedly alter edge uniformity and the formation of local minima near electrode edges. Accurate interpretation of ion dynamics is crucial because it affects the density and potential profiles and is indispensable for analyzing asymmetric configurations.

## 4. Discussion

The effectiveness of a fluid model and the necessity of PIC simulations for studying LTPs have been reviewed. Quantitative evaluations have been performed using fundamental kinetic theory and a PIC code, comparing with the HPEM fluid code. The kinetic analysis, based on both theory and simulations, clearly reveals inherent approximations and discrepancies in fluid analysis arising from misinterpretations of EEPFs. The results demonstrate that PIC simulations are indispensable tools for achieving an authentic understanding of kinetic effects.

Section 2 presented a detailed process for deriving the fluid approximations used in fluid codes. The primary concept behind these approximations is to predefine the distribution function as quasi-Maxwellian (e.g., Druyvesteyn, bi-Maxwellian) or as linear combinations thereof, assuming that collisions dominate in the plasma. These approximations substantially simplify the fluid equations, making numerical calculations feasible. However, in LTPs, the effects of electric fields can predominate, causing the distribution to deviate from the quasi-Maxwellian shape. Strong electric fields are observed in the sheath near the plasma-wall interface, a key parameter in most LTPs. Due to simplifications in EEPFs, fluid codes can yield non-negligible discrepancies, particularly at low pressures and in asymmetric electrode geometries. Field-induced non-Maxwellian effects, which are not captured by the fluid approach, include the generation of energetic particles, nonlocal transports of particle and heat fluxes, and viscosity in sheath regimes. The theoretical analysis is validated using PIC simulations, which are compared with fluid simulations. Detailed simulation results for symmetric and asymmetric geometries were presented in Section 3, where discrepancies between PIC and fluid simulations were demonstrated and analyzed from a kinetic perspective.

The observed ion pressure anisotropy suggests that the isotropic pressure closure commonly used in fluid models is inadequate for asymmetric low-pressure discharges, even when the full momentum equation is solved. Addressing this limitation within a fluid framework requires higher-order moment closures that evolve the full pressure tensor. Recent advances include the ten-moment multi-fluid model, which solves for all six independent components of the symmetric pressure tensor [54,55], and quadrature-based moment methods such as HyQMOM, which have been shown to reproduce kinetic ion transport in bounded plasmas with near-PIC fidelity [56]. However, these advanced closures have so far been applied primarily to magnetized plasmas and one-dimensional bounded systems; their systematic extension to multidimensional RF discharge geometries remains an open challenge. The present PIC results, which quantify the magnitude and spatial structure of ion pressure anisotropy in a representative CCP configuration, provide a benchmark dataset for validating such advanced fluid closures in future work.

The ion energy distribution functions (IEDFs) are primarily measured on the substrate rather than in the bulk because they are important for materials processing [57]. The bulk plasma has a Maxwellian distribution at very low temperatures (less than 0.04 eV). In the presheath, the IEDF begins to exhibit a drift term, which becomes exaggerated in the sheath, and the distribution is no longer Maxwellian. Dual-frequency CCPs have been well investigated for the independent control of IEDFs and ion flux [57].

Even in idealized geometries simpler than a real processing chamber, critical discrepancies are observed. Given that non-Maxwellian effects, reproduced only by PIC simulations, play crucial roles in most LTP scenarios, PIC simulations are imperative for LTP analyses. However, in certain cases, the fluid model remains effective to simulate LTP. The primary challenge in using the PIC code is the long computation time, which is attributed to the large number of superparticles, even when employing multi-CPU parallelization. However, this limitation has been mitigated by the development of modern GPUs. GPU acceleration has enabled 2D PIC codes to achieve competitive speed with multi-CPU 2D fluid codes. While GPU-accelerated PIC simulations are highly effective for analyzing LTP, it is crucial to emphasize that fluid simulations offer distinct advantages and remain valuable tools. A fluid model can handle complex gas chemistry without requiring cross-section data. Additionally, advanced fluid codes can handle more complex 3D geometries using the finite element method (FEM) or the finite volume method (FVM). They can accommodate a larger number of species and reactions. Therefore, PIC, fluid, and their hybrid schemes should be collaboratively developed and used to study LTPs efficiently.

## 5. Conclusions

We have examined the effectiveness of fluid models and the necessity of kinetic PIC simulations for analyzing low-temperature RF-CCPs by combining kinetic theory with two-dimensional simulations in symmetric and asymmetric geometries. Fluid simulations can reproduce global quantities such as the electron density and plasma potential in geometrically symmetric reactors, but they fail to capture the nonlocal electron temperature profiles and the strongly non-Maxwellian electron energy probability functions that appear at low *pd*.

In asymmetric configurations that more closely resemble realistic process chambers, the PIC simulations showed strong spatial nonuniformities in electron density and temperature, energy-dependent electron trapping, and local potential wells, all absent in the fluid results, highlighting the limitations of assuming local transport and quasi-Maxwellian distributions in such regimes. Most notably, the full ion pressure tensor obtained from PIC exhibits substantial anisotropy, with off-diagonal components reaching up to 10% of the diagonal terms in the bulk plasma, leading to diffusion forces that cannot be represented by isotropic pressure gradients alone. In contrast, the electron pressure tensor remains nearly isotropic due to efficient collisional relaxation, supporting the continued use of isotropic closures for electrons while calling for more sophisticated treatments of ion viscosity in asymmetric low-pressure plasmas.

A clear modeling strategy is that fluid simulations remain appropriate for higher-pressure, geometrically symmetric discharges with short electron energy relaxation lengths. In contrast, PIC approaches are required for low-pressure, strongly asymmetric reactors in which nonlocal kinetics and pressure anisotropy are significant.

## Figures and Tables

**Table 1:** Numerical conditions for PIC and HPEM simulations.

Parameter	Value
Frequency	13.56 MHz
Voltage	100 V
Pressure	100 or 500 mTorr
Gas	Argon
Gap distance	2–3 cm (Symmetric)
Sidewall gap	0.8–3 cm (Asymmetric)
Grid size	200 μm (PIC) vs. 500 μm (HPEM)
Time step	14.4 ps (PIC) vs. 184 ps (HPEM)
Superparticle weight (np2c)	2 × 10^6^
Steady-state convergence	≤2000 RF cycles

## Data Availability

The original contributions presented in the study are included in the article, further inquiries can be directed to the corresponding author.
